# Responsive Plasmonic Nanomaterials for Advanced Cancer Diagnostics

**DOI:** 10.3389/fchem.2021.652287

**Published:** 2021-03-18

**Authors:** Rong Lu, Jiankun Ni, Shengnan Yin, Yiding Ji

**Affiliations:** Suzhou Ninth People’s Hospital, Suzhou, China

**Keywords:** responsive nanostructures, cancer, sensing, detection, imaging

## Abstract

Plasmonic nanostructures, particularly of noble-metal Au and Ag, have attracted long-lasting research interests because of their intriguing physical and chemical properties. Under light excitation, their conduction electrons can form collective oscillation with the electromagnetic fields at particular wavelength, leading to localized surface plasmon resonance (LSPR). The remarkable characteristic of LSPR is the absorption and scattering of light at the resonant wavelength and greatly enhanced electric fields in localized areas. In response to the chemical and physical changes, these optical properties of plasmonic nanostructures will exhibit drastic color changes and highly sensitive peak shifts, which has been extensively used for biological imaging and disease treatments. In this mini review, we aim to briefly summarize recent progress of preparing responsive plasmonic nanostructures for biodiagnostics, with specific focus on cancer imaging and treatment. We start with typical synthetic approaches to various plasmonic nanostructures and elucidate practical strategies and working mechanism in tuning their LSPR properties. Current achievements in using responsive plasmonic nanostructures for advanced cancer diagnostics will be further discussed. Concise perspectives on existing challenges in developing plasmonic platforms for clinic diagnostics is also provided at the end of this review.

## Introduction

Plasmonic nanomaterials have attracting long-lasting attentions due to their unique localized surface plasmon resonance (LSPR) under light excitation (Wang et al., 2007; Fang and Zhu, 2013). In LSPR, the resonant oscillation of free electrons is localized on particle surface, forming localized surface plasmon, and features tunable extinction at the resonant wavelength (Athukorale et al., 2019). In principle, the LSPR strength and peak position are determined by many factors, including chemical components, sizes, shapes, and surrounding dielectric properties (McFarland and Van Duyne, 2003; Jain et al., 2007; Bonatti et al., 2020). Therefore, chemists have developed many elegant synthetic methods for preparing plasmonic nanostructures with defined structures and tunable LSPR properties (Jones et al., 2011; Rycenga et al., 2011). Depending on the morphology and size of the plasmonic nanostructures, their LSPR can be tuned from visible to infrared regions. The widely accessible resonant wavelength provides unlimited opportunities in light-related applications. A few important examples are colorimetric sensing, spectroscopic detection, optical devices, photocatalysis, and biomedicine (Stewart et al., 2008; Kawata et al., 2009; Guerrini and Graham, 2012; Abu-Thabit and Ratemi, 2020).

In addition to the physical properties of the materials, the LSPR of plasmonic nanostructures are also responsive to the dielectric properties of surrounding environments. For example, when the refractive index of environment is changing, their LSPR peaks shifts dynamically. This property has been extensively studied for determining refractive index by carefully measuring the peak positions. When plasmonic nanoparticles assemble into a considerably close manner, the LSPR will couple mutually, forming a coupled plasmon resonance (Jones et al., 2011). The coupling strength and peak position are determined by several key parameters, including interparticle distances, assembled structures, and size of the superstructures (Sönnichsen et al., 2005; Jain and El-Sayed, 2010). Tuning these parameters lead to active plasmonic nanostructures. If plasmonic nanostructures have an anisotropic shape, the LSPR is also determined by the structural orientation (Wang and Yin, 2016). A noticeable example is Au nanorods that have transverse and longitudinal mode with two distinct LSPR peaks in their spectra. These responsive plasmonic nanostructures represent important progresses in materials science and have provided advanced materials for practical applications, particularly in cancer diagnosis (Bellassai et al., 2019). In this minireview, we aim to summarize recent research progresses in creating responsive plasmonic nanostructures. We elaborate the dependence of LSPR properties of responsive plasmonic structures on surrounding dielectrics, coupling distances, and particle orientation. Several emerging colloidal synthesis methods and assembly approaches are discussed. Their promising applications in cancer diagnosis are provided with specific focus on biosensing and imaging. Perspectives and existing challenges are summarized at the end of this review.

## Responsive Plasmonic Nanostructures

**FIGURE 1:**
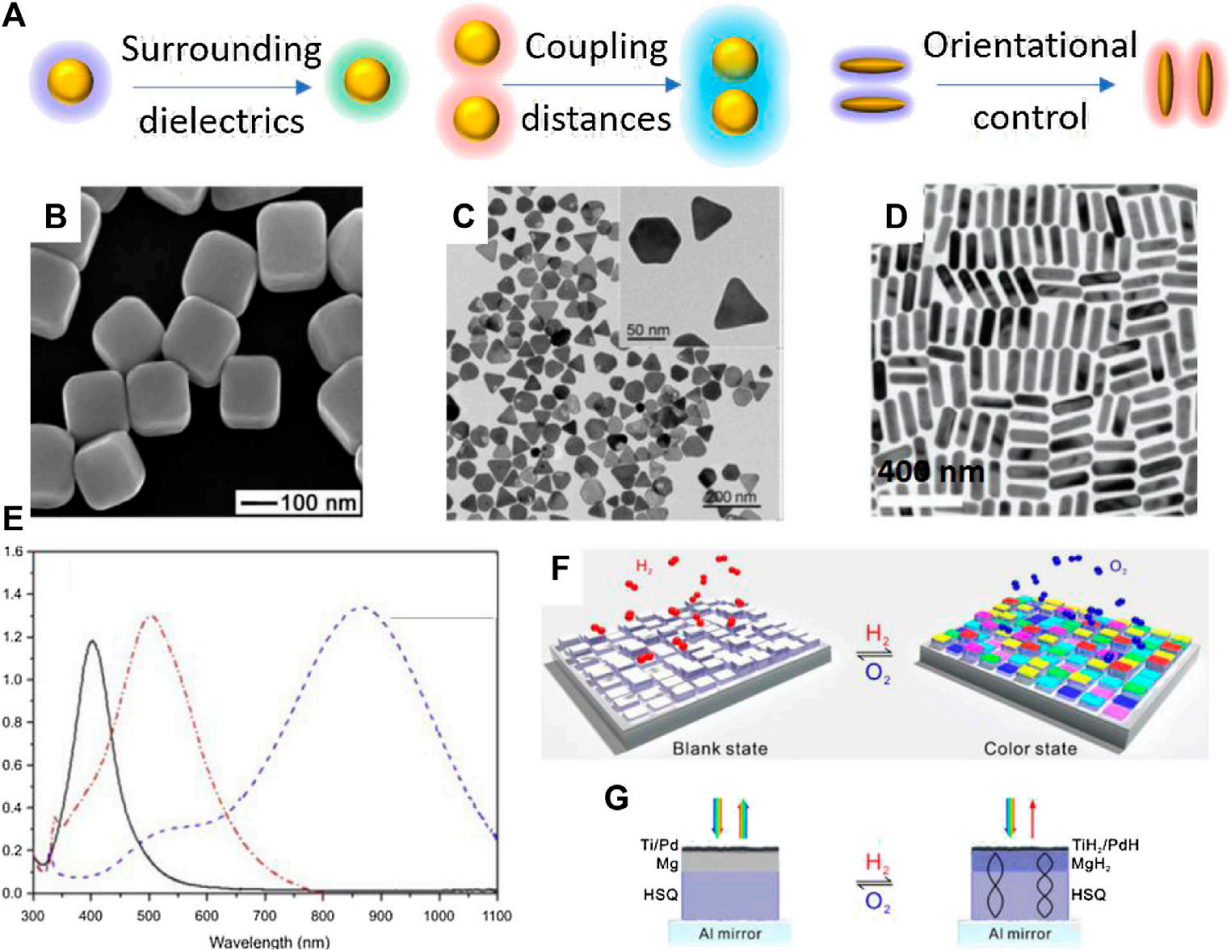
Representative plasmonic nanostructures of single component. **(A)** Schematic illustration of responsive plasmonic nanostructures under external stimuli. **(B)** Scanning electron microscopy (SEM) image of Ag nanocubes. Reproduced from Sun and Xia (2002) with permission from American Association for the Advancement of Science **(C)** Transition electron microscopy (TEM) image of Ag@Au nanoplates. Reproduced from Gao et al. (2012) with permission from Wiley-VCH Verlag GmbH & Co. KGaA, Weinheim. **(D)** TEM image of Au nanorods. Reproduced from Ye et al. (2012) with permission from American Chemical Society. **(E)** Absorption spectra of Ag nanoparticles with different shapes. From left to right: nanospheres, nanodiscs, and triangular nanoplates. Reproduced from Cheon et al. (2019) with permission from Dove Medical Press. **(F)** The scheme of dynamic plasmonic displays in response to chemicals. **(G)** Working mechanism of the color changes in dynamic plasmonic displays. Reproduced from Chen et al. (2017b) with permission from American Chemical Society.

These exists several strategies for preparing responsive plasmonic nanostructures (Figure 1A), including tuning the surrounding dielectrics, controlling the orientation of anisotropic structures, and tuning the coupling distances of assembled nanostructures.

### Tuning the Surrounding Dielectrics

Tuning the surrounding dielectrics of plasmonic nanostructures is a simply yet effective way to alter the plasmonic peak position and perceived colors. This strategy uses chemical reactions or the specific binding between plasmonic nanomaterial surface with various molecules, such as ligands, proteins, DNA, and functional polymers, to prepare responsive plasmonic nanostructures. Because of the easy surface functionalization of metallic nanoparticle, widely accessible functional molecules and plasmonic nanostructures, including nanocubes (Figure 1B) (Sun and Xia, 2002), nanoplates (Figure 1C) (Gao et al., 2012), and nanorods (Figure 1D) (Ye et al., 2012), this strategy have been extensively studied in chemical sensing and biological detections. Notably, the shapes of plasmonic nanostructures determine the LSPR properties of the structures. For example, Ag nanostructures with different shapes have widely tunable LSPR position from visible to near infrared region (Figure 1E) (Cheon et al., 2019). Generally, an increase in dielectric constant or refractive index leads to a redshift of the LSPR peak of the plasmonic nanostructures (McFarland and Van Duyne, 2003; Rindzevicius et al., 2007; Shen et al., 2013). Compared with conventional electrical sensors, plasmonic sensors are cheaper, easier to use while providing comparable sensitivity. To create detectable color changes or LSPR peak shift, plasmonic nanostructures are functionalized by active molecules or embedded in polymer matrix or functional substrates, which can react with target molecules or exhibit phase changes under external stimuli. In practical applications, several important external stimuli include heat, light, electric fields, magnetic fields, chemicals. For example, Chen et al. developed dynamic displays based on the active plasmonic color changes in response to chemicals (Chen et al., 2017b). The display exhibits brilliant plasmonic colors in exposure to hydrogen (Figure 1F). Interestingly, the color can be readily erased by exposure to oxygen. Fabricated by nanolithography, each pixel comprises an Al minor, a hydrogen silsesquioxane (HSQ) layer, a Mg layer, and a top Ti/Pd layer (Figure 1G). The pixel is in gray scale right after nanolithography. Once being exposed to hydrogen, magnesium undergoes unique metal to dielectric transitions, giving rise to vivid color changes on each pixel (Figure 1G). Thermally responsive plasmonic nanostructures can be prepared by using the temperature-dependent phase transition of polymers, liquid crystals metals and metal oxides. A noticeable example is Poly(N-isopropylacrylamide) (PNIPAM), which has reversible phase transition from hydrophilic swollen phase to hydrophobic collapsed phase when heated in water above a lower critical solution temperature (LCST at 32°C). Therefore, if PNIPAM was grafted on Au nanoarrays, this phase transition will induce an increase of 0.6 in refractive index and 10-nm redshift of the LSPR peaks. Similarly, it is possible to prepare light-activated responsive plasmonic nanostructures by introducing photochromic molecules as matrix. Under light irradiation, the photochromic molecules switch between two thermodynamically stable states via photochemical reactions. Molecular reactions that have been broadly used in practice include cis-trans transition (e.g., azobenzene), photon-initiated polymerization and dissociation, photo-cyclization (Sasaki and Nagamura, 1997; Ming et al., 2010; Pardo et al., 2011; Joshi et al., 2014). Particularly, azobenzene has cis-to-trans transition under 450-nm light excitation, and researchers have revealed that it can induce a ∼21-nm peak redshift of Au nanoprisms. A close investigates demonstrated an increase of ∼0.5 nm in the height of grafted azobenzene molecules, which contributed to the increases in refractive index and the LSPR redshift (Joshi et al., 2014). In addition to these physical stimuli, chemical reactions have also been extensively used in preparing responsive plasmonic nanostructures. Tracking the LSPR shift and colorimetric responses are an established approach to various chemical sensors. Typically, chemical stimuli include humidity, pH, electrochemical reactions, ionic strength, and biological molecules (Gao et al., 2012; Byers et al., 2015; Xiong et al., 2016). The semiconducting polyaniline has great variations in its conductivity and dielectric properties upon protonation. Therefore, the LSPR peak of Au nanorods that are coated with polyaniline has reversible shift between 640 and 740 nm during protonation-deprotonation cycles (Lu et al., 2017). Gao et al. developed a highly sensitive biological sensor based on Ag nanoplates (Figure 1C). A thin layer of Au was coated on Ag to enhance chemical stability of Ag under various etching environments. They demonstrated superior performance of Ag nanoplates in detecting proteins (Gao et al., 2012).

### Orientational Control

Plasmonic nanostructures with anisotropic shapes, such as nanorods and nanoplates, have orientation-dependent plasmonic excitations. That is, the excitation of free-electron oscillation is highly dependent on the structural orientation relative to the light polarization. Taking Au nanorods for example, they have transverse and longitudinal LSPR modes (Wang and Yin, 2016). When the short and long axis of the Au nanorods are parallel to the light polarization, only transvers and longitudinal modes will be excited, respectively. Therefore, controlling the orientation of anisotropic plasmonic nanostructures enables active tuning of the LSPR peak intensity. The key is to develop a reversible, remote, and fast method to control the collective orientation of plasmonic nanostructures. Early researches employed anisotropic plasmonic nanostructures deposited on substrates for measuring their orientation-dependent plasmonic excitations (Tabor et al., 2009; Chang et al., 2010). While they provided precise prediction of physical properties, their further advancement in practical applications is limited by the presence of the bulk substrates. To this end, recent progress focused on new techniques for controlling the structural orientations, including electric fields, magnetic fields, and mechanical forces (Zhang et al., 2019).

**FIGURE 2:**
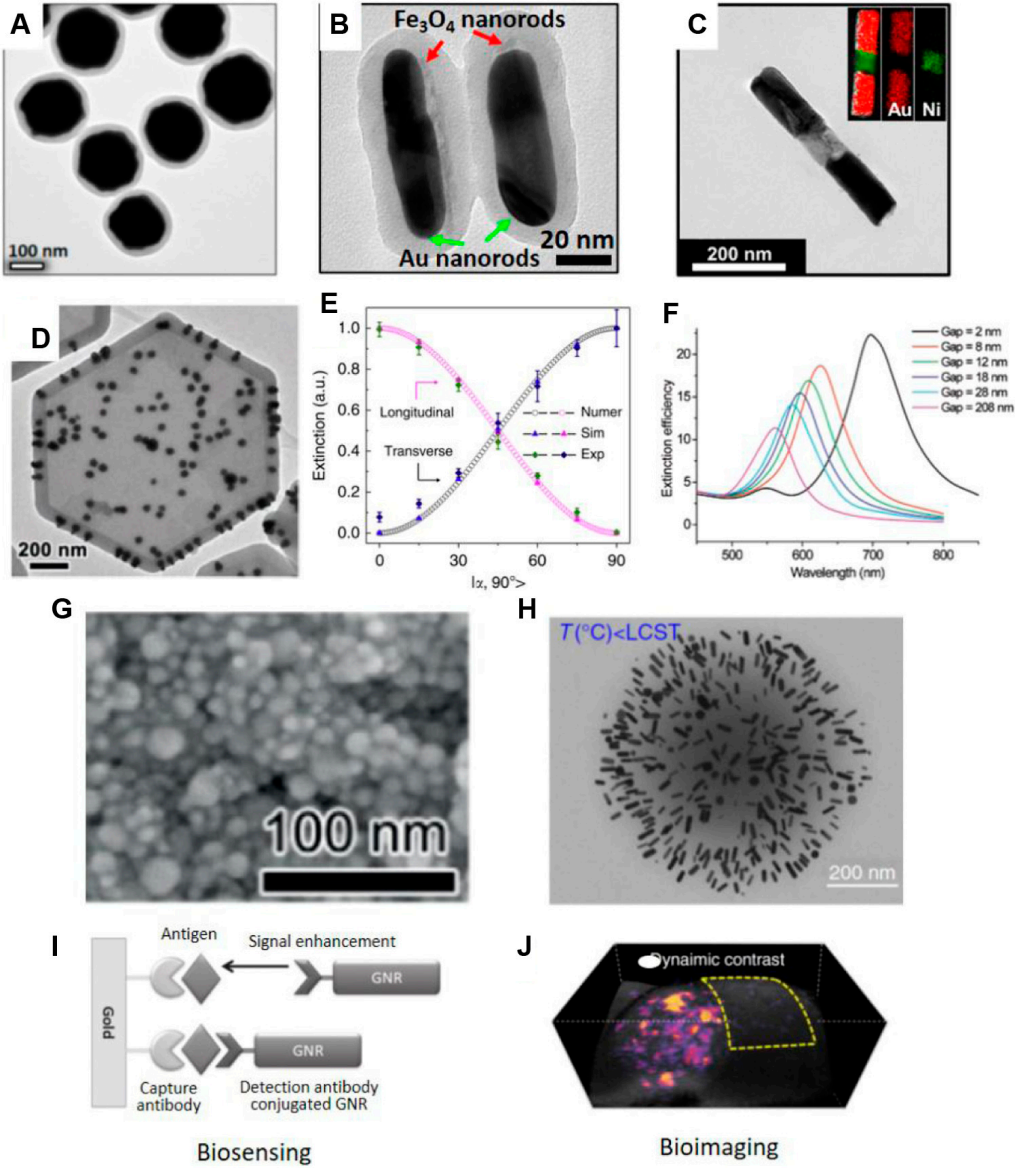
TEM images of **(A)** Fe_3_O_4_@Au@polymer nanospheres. Reproduced from Li et al. (2020a) with permission from American Chemical Society. **(B)** Fe_3_O_4_/Au@polymer nanorods, Reproduced from Li et al. (2020b) with permission from the authors. **(C)** Au-Ni-Au multi-segment nanorods. Reproduced from Jung et al. (2018a) with permission from American Chemical Society. **(D)** Au dimer on magnetic nanoplate hybrid structure. Reproduced from Feng et al. (2019) with permission from Wiley-VCH Verlag GmbH & Co. KGaA, Weinheim. **(E)** Dependence of plasmon peak intensity on the orientation of the Fe_3_O_4_/Au@polymer nanorods. Reproduced from Li et al. (2020e) with permission from the authors. **(F)** Calculated extinction efficiency spectra of Au nanostructures with different separations. Reproduced from Jain et al. (2007) with permission from American Chemical Society. **(G)** SEM image of Ag nanoparticles deposited on substrates. Reproduced from Liu et al. (2019) with permission from Wiley-VCH Verlag GmbH & Co. KGaA, Weinheim. **(H)** TEM images of PNIPAM-Au nanorods nanocomponents prepared at temperature below LCST. Reproduced from Chen et al. (2017a) with permission from the authors. **(I)** Biosensing. Reproduced from Law et al. (2011) with permission from American Chemical Society. GNR represents Au nanorods. **(J)** Bioimaging. Reproduced from Chen et al. (2017a) with permission from the authors.

The first example is electrically responsive plasmonic nanostructures. They were prepared by dispersing anisotropic plasmonic structures in conventional liquid crystals. The interactions between the nanostructures and the liquid crystal molecules can regulate the collective orientation of the guest nanostructures. Therefore, simply changing the applied electric field alters the structural orientation so that their LSPR intensity can be actively tuned (Liu et al., 2014a; Rozic et al., 2017). This strategy strongly relies on the interactions between molecules and nanostructures, which, in combination with the presence of the host liquid crystals, hinders their practical use in chemical and biological sensors. Instead, magnetically responsive plasmonic nanostructures have attracted great attentions recently due to the fast, remote, and fully reversible magnetic orientational control (He et al., 2012; Li and Yin, 2019; Li et al., 2020d). Magnetic nanostructures with anisotropic shapes, like Fe_3_O_4_ nanorods, nanoplates and Ni nanorods, have preferred alignment in responsive to an external magnetic field. To minimize the magnetization energy, their long axes will be parallel to the external magnetic field (Goebl et al., 2016; Feng et al., 2019; Chen et al., 2020). Therefore, it is possible to actively tune the orientation of plasmonic nanostructures if the magnetic and plasmonic anisotropy is coupled in a defined anisotropic structure. Surface attachment has been demonstrated as an easy approach to Fe_3_O_4_/Au hybrid nanorods (Wang et al., 2013). The LSPR intensity and the perceived color of Au nanorods in a colloidal solution can be quickly tuned by applying a magnetic field. A few advanced techniques enable direct colloidal synthesis of magnetic-plasmonic hybrid nanostructures, including Fe_3_O_4_@Au core/shell nanospheres (Figure 2A) (Li et al., 2020a), Fe_3_O_4_/Au nanorods (Figure 2B) (Li et al., 2020b), Fe_3_O_4_/Ag nanorods (Li et al., 2020e), Au/Ni/Au (Figure 2C) (Jung et al., 2018a), Au/Fe/Au multiblock nanorods (Jung et al., 2018b), and Au dimer on magnetic nanoplate hybrid structure (Figure 2D) (Feng et al., 2019). One advantage of magnetic orientational control is the selective excitation of the two modes of Au nanorods. Figure 2E shows the orientation-dependent plasmonic excitation of the transverse and longitudinoal modes. It demonstrates that, under polarized-light excititation, only transverse or longitudinal modes will be excited when the rod alignment is perpendicular or parallel to light polarization, respectively.

### Tuning the Coupling Distances

When plasmonic nanostructures self-assembled, their LSPR will couple with each other (Sönnichsen et al., 2005). The plasmon coupling induces remarkable LSPR peak shift and color changes, which is determined by the interparticle distances (Li et al., 2020c). As shown in the calculated spectra in Figure 2F, gradually decreasing the gaps between two Au nanodiscs from 208 to 2 nm leads to a significant redshift of the coupling peaks (Jain et al., 2007). This close measurement produces the important plasmonic rule equation to accurately calculate the coupling peak position. For Au particle pair in protein medium, its derived equation can be expressed as Δ*λ*/*λ* = 0.18*exp(-(*s*/*D*)/0.23). In this case, Δ*λ*/*λ* is the fractional peak shift, *s* is the interparticle edge-to-edge separation, and *D* is the particle diameter. Therefore, tuning the coupling distances between plasmonic nanostructures have been extensively explored for spectroscopic detection. And the highly localized electric fields between coupled plasmonic nanostructures can greatly enhance the sensitivity of surface-enhanced Raman scattering (Porter et al., 2008). There exist several well-defined systems to prepare responsive plasmonic assemblies with tunable optical properties. They can be developed by either direct assembling plasmonic nanoparticles in colloidal dispersions or by perturbating the assembled structures in soft matrix under physical and chemical stimuli (Ding et al., 2016; Liu et al., 2018; Ding and Baumberg, 2020). For example, plasmonic nanochains can be directed assembled in colloidal dispersions by regulating the interactions between nanoparticles. This can be achieved by adjusting ionic strength, solvation, pH, and temperature of the assembly solutions (Zhang and Wang, 2008; Hu et al., 2016). In 2014, Liu et al. developed thermo-responsive assembly of charged Au nanoparticles and achieved reversible tuning of the plasmon coupling between them (Liu et al., 2012). This work was based on the prediction that the electrostatic interactions between charged nanoparticles can be regulated by solution temperature, in addition to salt concentrations. Using the assembled Au nanochains as building blocks, the authors further developed a pressure sensor (Han et al., 2014). The mechanochromic sensor had a soft polymer matrix and assembled Au nanochains inside. Due to the plasmon coupling between neighbouring Au nanoparticles, the film had an initial blue color. Pressing the film enlarges the interparticle distances, leading to the decoupling between Au nanoparticles. In the spectra, they observed a gradual disappearance of the coupling LSPR peaks at long wavelength, which exhibited colorimetric responses from blue to red. Most recently, Liu et al. developed an ultra-sensitive color switching of plasmonic Ag films. Poly (acrylic acid) (PAA) capped Ag nanoparticles were deposited on solid substrates under the presence of sodium borate slats (Liu et al., 2019). The solid film contains densely packed Ag nanoparticles with close contact (Figure 2G). The plasmon coupling between Ag nanoparticles has a LSPR peak at 526 nm so that the plasmonic film was pink. The borate will hydrolyze rapidly in response to moisture and produce OH^−^ ions, which subsequently deprotonate the PAA on Ag nanoparticles, increase the surface charge and interparticle distances, weaken the plasmonic coupling and finally lead to a blue shift of the LSPR peaks. Notably, this process occurs in the solid film within 0.3 s. And the film exhibited drastic color changes in response to trace amount of humidity, such as humidity from breathing and fingertip. With considerable cycling performances, this film is expected to have great potentials in developing touchless biological sensors or high-performance anticounterfeiting devices. Chen et al. reported a temperature-dependent plasmonic responses of Au nanorods (Chen et al., 2017a). To prepare the stimuli-responsive plasmon coupling, Au nanorods were incorporated into PNIPAM nanogels. When the temperature is below the LCST, the PNIPAM nanogels are hydrophilic swollen state with expended volume (Figure 2H). When the temperature increases to above the LCST, the nanogels become hydrophobic so that the volume will reduce. In the swollen state, the distance between adjacent Au nanorods is large and the Au nanorods feature a sharp, strong LSPR peak. At high temperature, the shrunk nanogels activate the plasmon coupling between Au nanorods, leading to LSPR peak shift. As a result, the initial peak intensity of Au nanorods decreased significantly. The nanogels are advantageous among the temperature-responsive systems in several aspects. Firstly, their responses are fast due to the quick heat transfer at the nanoscale. Secondly, the small size of nanogels is ideal for *in vivo* biological detections and imaging. Thirdly, many plasmonic nanostructures can be incorporated into the nanogels so that it provides many opportunities for preparing smart optical devices and active contrast agents.

## Applications in Cancer Diagnosis

The three strategies to prepare responsive plasmonic nanostructures are promising in cancer diagnosis, particularly in cancer detection and imaging (Cheng et al., 2014). For example, plasmonic nanostructures are extensively used as enhancers to improve the sensitivity of detection and imaging. This technique is based on their LSPR shift as a result of surrounding dielectric changes and/or coupling distance changes when in recognition of biological molecules (Mejía-Salazar and Oliveira, 2018). Most recently, orientational control has also been used for detecting biological species (Hilal et al., 2020). In this section, we elucidate a few emerging applications of responsive plasmonic nanostructures in cancer diagnosis.

### Sensing and Detections of Cancer Biomarkers

The responsive plasmonic nanostructures are ideal colorimetric sensors for detecting biological species, such as cancer biomarkers (Laing et al., 2017; Mejía-Salazar and Oliveira, 2018). They provide perceptible color changes in response to the presence of target molecules, without additional energy consumption and readouts. Spectroscopic detection is also possible to provide accurate and sensitive estimation of the concentrations of target species. These advantages enable non-invasive and point-of-care cancer diagnosis with low detection limits. Therefore, many plasmonic biosensors have been developed for fast, label-free, accurate, and real-time detection of cancer cells and biomarkers. Some biosensors use the LSPR peak shifts of plasmonic nanostructures in recognition of target molecules for detection while other biosensors use the surface-enhanced spectroscopic techniques, including SERS, surface-enhanced infrared absorption spectroscopy, and surface-enhanced fluorescence (Kinkhabwala et al., 2009; Fayyaz et al., 2012; Taylor and Zijlstra, 2017). Figure 2I shows a representative working principle of plasmonic immunoassay using Au nanorods as the responsive plasmonic nanostructures. In this work, Law et al. developed a plasmonic biosensors for the detection of cancer biomarkers. Specifically, a plasmonic film and Au nanorods were modified with antibody, which was used as ultrasensitive immunoassay. They found that the Au nanorods could enhance the signals of tumor necrosis factor alpha antigen by 40 times. Such a significant enhancement in sensitivity was contributed to the near-field coupling between solid plasmonic film and the Au nanorods under the antibody-antigen recognition (Law et al., 2011). This work represents practical use of actively tuning the coupling distance between plasmonic nanostructures for high-performance detection for biological species. In enhanced spectroscopy, Raman or fluorescence molecules are usually coupled with the responsive plasmonic nanostructures (Vo-Dinh et al., 2010). Under this scenario, the excitation of these scattering or emissive molecules will be greatly enhanced by the localized electric fields (Li et al., 2017; Wu et al., 2018). Since the localized field strength is dependent on the interparticle separation and the coupling strength of plasmonic nanostructures, the SERS or fluorescence signals can therefore be tuned by external stimuli. Several common practices involve responsive interactions between plasmonic nanostructures or transformation of functional molecules that are grafted on particle surface. More specifically, a few well-established working principles induce conformal changes of molecules, specific interactions between biological molecules, hydrogen bonding and tunable electrostatic/steric interactions (Dasary et al., 2009; Qian et al., 2009; Guerrini et al., 2012).

### Advanced Cancer Bioimaging

The LSPR of plasmonic nanostructures have two physical effects. It can scatter light at the resonant wavelength or absorb light to convert to heat at the same resonant wavelength. Because of the photothermal effects, plasmonic nanostructures also serve as a good candidate for photoacoustic (PA) imaging (Li et al., 2014). In PA imaging, the temperature increase induced by the plasmonic nanostructures induces acoustic wave in biological tissues and cancers, which is detected to form 2D or 3D images (Liu et al., 2014b; Song et al., 2014; Zeng et al., 2019). PA imaging has higher resolution and better tissue penetration compared with conventional optical imaging, like fluorescent imaging, due to the weaker scattering of acoustic waves than that of electromagnetic waves in biological tissues (He et al., 2013; Liu et al., 2014b). Responsive plasmonic nanostructures have many advantages compared with other PA contrast agents, such as good biocompatibility, easy surface functionalization for targeting, and most importantly active PA signals in response to remote, external stimuli. Their ability to actively alter PA signals represents open platforms for several advanced imaging techniques. The characteristic of responsive plasmonic nanostructures is to actively adjust their LSPR and photothermal properties under external stimuli. Therefore, it is possible to derive more information from the dynamic signals. Background-free PA imaging is one successful use of these unique properties for cancer diagnosis. In a common practice, the PA signals from the active contrast agent varies significantly and regularly depending on the stimuli, such as temperature and light, while the PA signals from endogenous molecules remain constant. In an imaging sequence, PA images will be acquired several times when external stimuli are applied so that the PA signals from the active contrast agents changes accordingly. A pixel subtraction is performed between two imaging frames, usually with highest and lowest PA intensities from the active contrast agents. Because the PA signals from backgrounds remains constant during PA imaging, they will be totally removed by the data processing. The differences between signals from active plasmonic nanostructures form a clear contrast without any background signals. A representative background-free PA image is shown in Figure 2J. In this 3D volume rendering, PA signals from tumor surroundings were removed completely, forming a high-specific imaging in tumor (Chen et al., 2017a). This advanced imaging technique is expected to have great potential applications due to its ability in enhancing the imaging contrast and specificity.

## Conclusion and Perspectives

This minireview summarizes recent research activities in preparing responsive plasmonic nanostructures. The working principles are discussed with specific focus on existing strategies for achieving active plasmonic properties in response to different stimuli. Noticeable examples are provided during the discussion, including recent achievements in direct colloidal synthesis of hybrid and multicomponent nanostructures. Due to their ability to alter their LSPR under external stimuli, they have been demonstrated as advanced materials for creating functional biosensors and active contrast agents for cancer diagnosis. Compared with other cancer nanomedicines, such as fluorescent probes, conducting polymers, or carbon-based materials, plasmonic nanomaterials have several merits. Firstly, the shape and size of plasmonic nanostructures can be easily controlled in colloidal synthesis, providing widely accessible LSPR peak positions. Secondly, plasmonic nanostructures, particularly of Au, have good biocompatibility. Thirdly, responsive plasmonic nanostructures are very sensitive to surrounding physical properties so that they represent an open platform to prepare smarts materials. Highly sensitive biosensing and background-free imaging are two convincing examples. Considering these advantages, researchers have developed many reliable approaches to responsive plasmonic materials, which play important roles in cancer detection and imaging. On the other hand, however, there exist several challenges in both creating responsive plasmonic nanostructured materials and their further developments in cancer diagnosis. From the aspect of colloidal synthesis, it requires additional efforts in developing approaches to hybrid nanostructures. Particularly, plasmonic hybrid nanostructures providing easy access to surface functionalization are important for their promising applications in cancer diagnosis. In the colloidal assembly of plasmonic nanostructures, the existing strategies provide plasmonic assemblies that are responsive to several key stimuli, like temperature and pH. To precisely control the interparticle separation is important in both preparation and practical applications. However, it is still difficult to accurately control the assembly kinetics and dynamics to produce plasmonic entities with uniform size, configuration, and hot spots. It is expected that templating approach is a promising method to overcome these long-last challenges. The use of a suitable nanostructured materials for templates can produce single-component, Janus or hybrid nanostructures, which are responsive to external stimuli. These methods are highly desirable for providing responsive plasmonic nanomaterials with small sizes, well-defined plasmonic properties, and readily accessible surfaces for functionalization, thus setting the stage ready for exploiting their exciting performances in cancan diagnosis.
